# EXAMINING MECHANISMS OF CHILDHOOD COGNITIVE CONTROL

**DOI:** 10.5334/joc.314

**Published:** 2023-08-25

**Authors:** Keertana Ganesan, Claire R. Smid, Abigail Thompson, Elisa S. Buchberger, Joshua Spowage, Somya Iqbal, Harriet Phillips, Nikolaus Steinbeis

**Affiliations:** 1 Division of Psychology and Language Sciences, UCL, London, WC1H 0AP, UK; 2 Max-Planck Institute for Human Development, Lentzeallee 94, 14195 Berlin, Germany

**Keywords:** Cognitive control, Cognitive control training, Executive functions

## Abstract

Childhood cognitive control is an important predictor for positive development, yet interventions seeking to improve it have provided mixed results. This is partly due to lack of clarity surrounding mechanisms of cognitive control, notably the role of inhibition and context monitoring. Here we use a randomized controlled trial to causally test the contributions of inhibition and context monitoring to cognitive control in childhood. Sixty children aged 6 to 9-years were assigned to three groups training either inhibition, context monitoring group or response speed using a gamified, highly variable and maximally adaptive training protocol. Whereas all children improved in the targeted cognitive functions over the course of training, pre-post data show that only the inhibition group improved on cognitive control. These findings serve as a first step in demonstrating the promise inhibition-based cognitive control interventions may hold.

Imagine going out for a meal with colleagues. After a long day at the office, you feel famished. Your food comes to your table before everyone else’s and you are able to stop yourself from taking a bite. How are you able to control your pre-potent response? On a daily basis, people need to control and direct their thoughts and actions. Also known as cognitive control, this term describes a set of processes that support flexible goal-directed behavior (Botvinick, Braver, Barch, Carter & Cohen, 2001; Botvinick & Braver, 2015). Childhood cognitive control is predictive of later life success and well-being (Blair & Razza, 2007; Clark, Pritchard, & Woodward, 2010; Bull, Espy, Wiebe, Sheffield, & Nelson, 2011; Moffitt et al., 2011) and as such its study occupies a key position in child development research. The importance of cognitive control for positive development coupled with increased neural plasticity during childhood (Bunge & Zelazo, 2006; Kolb & Gibb, 2011; Wass, Porayska-Pomsta, & Johnson, 2011) has made it a primary target for interventions (Wass, Scerif, & Johnson, 2012). However, the precise mechanistic targets are still debated. Inhibition has long occupied a prominent role in cognitive control (Aron, 2007). More recently however it has been suggested that this can instead be subsumed by other cognitive processes, notably context monitoring (Chatham et al., 2012, Chevalier, Chatham, & Munakata, 2014; Hampshire, Chamberlain, Duncan & Owen, 2010; Sharp et al., 2010). Understanding the mechanisms constituent of cognitive control is key to optimize interventions aimed at improving this critical life skill. To examine the causal role of inhibition and context monitoring in cognitive control during childhood we used a 6-week training protocol, testing for the effects of training on several indices of cognitive control. We show that both inhibition and context monitoring improved during the course of training, but that only inhibition led to changes in several indicators of cognitive control.

Inhibition has long been considered to be at the core of cognitive and behavioral control (Aron, 2007). Factor analyses of executive function in middle childhood have consistently yielded factor loadings of inhibition (Messer et al., 2018, St Clair & Gathercole, 2006, Wu et al., 2011, Hartun et al., 2020), which in turn has been underpinned by a circumscribed neural network of brain regions including right inferior frontal gyrus (Aron et al., 2003). More recently it has been argued that the ability to inhibit unwanted thoughts or actions depends as much on monitoring the environment for contextual cues that indicate the need to change action (Chatham et al., 2012; Dodds, Morein-Zamir, & Robbins, 2011; Hampshire et al., 2010). Evidence in support of this view comes from tasks matched on context-monitoring but with different motoric demands (e.g., requiring a double key press instead of inhibition in response to signal). In adults, it was demonstrated that multiple neural and behavioural signatures of response inhibition tracked monitoring demands more closely than motoric-stopping demands, and behavioral measures of context-monitoring efficacy, but not stopping efficacy, predicted both response inhibition performance and associated rIFG activation (Chatham et al., 2012). According to this account, inhibition can be subsumed by a more general process of action selection (i.e. selecting between initiation and inhibition of action).

This revised account has not remained unchallenged (Aron, Robbins, & Poldrack, 2014). It has been argued that any infrequent stimulus (as is the case in virtually all studies arguing for a context monitoring account) require some form of inhibition (Aron et al., 2014). Further, the literature on Pavlovian response biases, where appetitive cues are inherently associated with Go responses and aversive cues with No-Go or Stop responses (Guitart-Masip et al., 2011), suggests that approach and avoidance (i.e. inhibition) are underpinned by fundamentally different processes. In sum, there remains substantial controversy over core processes of cognitive control. This controversy finds itself also in the developmental literature, where standard views of the primacy of response inhibition in cognitive control (Diamond, 2002) contrast with more recent accounts advocating for a core role of context monitoring (Winter & Sheridan, 2014; Chevalier et al., 2014). Given the importance of understanding the core processes of cognitive control in order to tailor interventions to foster this crucial skill early in life, causal evidence is needed.

A recent study in 7- to 9-year old children practicing either stopping an ongoing action or monitoring for cues that signalled the need to ‘go-again’ showed that practicing either activity improved response inhibition scores, but that children who had practised monitoring outperformed the inhibition group (Chevalier et al., 2014). However, this study only looked at the effects of different instructions after practice, rather than behavioural change, and failed to investigate how this could transfer to independent pre-post measures. Group differences could therefore also be attributed to pre-existing individual and task-related differences. As such, causal evidence for a unique role of context monitoring in cognitive control is still lacking. To remedy this, we used a pre-post design looking at the effects of inhibition training and context monitoring training on several indicators of cognitive control. We further included a control group training in response speed.

Training studies offer considerable leverage for causal inference on the involvement of key mechanisms (Chatham et al., 2012; 2014; Chevalier et al., 2014; Knoll et al., 2016). While working memory and cognitive flexibility have received most empirical attention, there is less work on the effects of inhibition training on cognitive control. This is in large parts due to early attempts proving unsuccessful leading to the premature conclusion that inhibition is too automatic a process to be trained (Cohen & Poldrack, 2008). More success at demonstrating the plasticity of inhibition has been shown recently using more adaptive training regimes (Zhang, Low, Gwynn, & Clemson, 2019; Zhao, Chen, Fu, & Maes, 2015; Delande et al., 2020; Berkman, Kahn, & Merchant, 2014, Verbruggen et al., 2013; Biggs et al., 2015). Moreover, a more fine-grained look at different types of cognitive control has been recommended (i.e. pro- and reactive control; Berkman et al., 2014; Prieto et al., 2023). While proactive control can be viewed as “early selection” in which goal-relevant information is actively maintained in a sustained manner before the occurrence of a cognitively demanding event, reactive control is activated as required, such as after the detection of a cognitively demanding event (Braver, Paxton, Locke & Barch, 2009; Braver, 2012). One recent study has shown that adults who had trained inhibition compared to a so-called sham training group exhibited neural activation patterns indicative of a shift from reactive to proactive cognitive control (Berkman et al., 2014).

In a pilot study, we examine the role of inhibition and context monitoring respectively in cognitive control. 60 children underwent a 6-week training of either inhibition, context monitoring or response speed, the latter of which served as a control for any generic training effects of inhibition and context monitoring. We hypothesised that training should lead to improvements in the targeted cognitive skill during the training. Further, we hypothesised differences between the three training groups on pre-post measures of cognitive control. We used behavioural indices of proactive control to further characterise the role of inhibition and context monitoring in cognitive control. We included an active control group training in response speed for which we did not expect any transfer onto measures of cognitive control.

## Methods

### Participants

Participants were children aged between 6.17 – 10.83 years (*M* = 8.25 years, *SD* = 0.87) from three different London schools. A convenience sample of 60 typically developing children (27 males, 33 females). Parental consent was obtained beforehand and the study was approved by the University College London research ethics committee. Children were tested onsite in a classroom by different researchers. Data collection occurred before and after the training. Training was delivered over a six-week period. Full pre-post data for our dependent variables was available from 57 participants for the Stop Signal Reaction Task (SSRT) and from 56 participants for the AX-Continuous Performance Test (AX-CPT). Across the schools, each child was randomly assigned to one of three groups: response inhibition, context monitoring, and response speed (Table 1).

**Table 1:** Training assignment of participants.


TRAINING GROUP	N	AGES (IN YEARS)	GENDER MALE (%)

Inhibition	19	8.84	42.11

Context Monitoring	21	8.37	55.00

Response speed	20	8.71	47.62


### General Procedure

During pre- and post-training test sessions, all participants were tested for approximately one-hour at their school, where they completed the behavioural tasks on a laptop. In the following 6-weeks, they participated in one or two training sessions per week with an experimenter in their school and were encouraged to engage in three additional training sessions at home, where they could access the same training games online.

### Training Games

For each group, the training games were presented in the same manner and with the same conceptual narrative, however the participant instructions varied according to the particular domains being trained. The overall narrative given to the participants was that they had crashed their plane in the desert. In order to return home, they will navigate through different four locations (i.e. forest, desert, snow, mountain), within which they must complete several of six individual games, enabling them to move to different locations on a map in order to meet a wise man who endows them with spare parts to fix their plane. After completing the first half of the games, they reach the wise man from whom they need to return to their plane.

Every session consisted of two games, and lasted for approximately 15 minutes. Number of trials and the number of required key presses differed per game, however the overall time spent playing within a session was equivalent across the different groups. The mechanistic aspect of the games differed across the different groups in the following ways: In the inhibition group, participants had to press the spacebar to respond to a go-signal, or refrain from pressing the spacebar when a stop signal appeared, essentially analogous to an SSRT. Games in this group used a staircase design that changed the Stop-Signal Delay (SSD) in steps of 50 ms according to the one-up-one-down procedure to achieve a 50% inhibition rate (Verbruggen & Logan, 2009; Logan et al., 1997), and was set to be 200 ms at the start. A successful stop trial would decrease the SSD by 50 ms, while an unsuccessful stop-trial would increase it by 50 ms. For the context monitoring group, participants had to press the spacebar in response to a go-signal and press the spacebar twice when presented with a ‘Double-go’ signal, similar to Chatham et al. 2012. The same staircasing procedure was used as in the inhibition group. For the response speed group, participants were simply instructed to press the spacebar as fast as possible. To make training adaptive for this group, a threshold was introduced that consisted of a rolling average of the response time of the previous ten trials plus two standard deviations. This ensured adaptive training mechanisms for all three training groups (for more details on the training, see *Supplementary Materials*).

### Motivation

At the end of each training session, participant could choose to complete a bonus game which was a shortened version of one of the six games, to get additional points. The choice to participate in bonus games was logged as a motivational measure. In addition, participants filled in a questionnaire regarding their motivation to participate in the training every week at school (available in *Supplementary Materials*). There was a total of 6 items on a 6-point scale (i.e. ‘Completely Agree’ to ‘Completely Disagree’). Negative items were reverse coded and a motivational score for each week was calculated by combining scores for the 6 items.

### Pre-Post Tasks

Pre-post measures on response inhibition and proactive control were collected as part of a bigger battery with other behavioural measures. Other measures (such as decision making indices) were collected as part of optimising them for a future study. As our study specifically focused on cognitive control, any other measures collected were excluded. This was collected before and after the 6-week training period.

#### Stop-Signal Response Task

As a measure of response inhibition, we used a modified and child-friendly version of the SSRT (Logan & Cowan, 1984; Matzke et al., 2018). Participants were instructed to press the spacebar as fast as possible when seeing a honey pot centrally located on the screen (i.e. go-trials). On 25% of the trials, a stop-signal (picture of bees) was presented with a variable delay (SSD) after the stop-signal. Participants were instructed to not press the spacebar if bees appeared after a honey pot (i.e. stop-trials). If participants did not respond after 600 ms, the honeypot disappeared. An intertrial-interval (i.e. fixation cross) was presented for 1250 ms before the presentation of the next trial. The task had a staircase design with changes in steps of 50 ms in the Stop-Signal Delay (200 ms) with a starting SSD of 200 ms. The SSD was then adjusted according to a tracking-procedure to achieve a 50% inhibition rate (Verbruggen & Logan, 2009), increasing the SSD by 50 ms after a successful stop trial and decreasing it by 50 ms after an unsuccessful stop-trial (one-up-one-down procedure, Logan et al., 1997). 10 practice trials were administered where feedback was provided, followed by the main task consisting of 60 go-trials and 20 stop-trials. No exclusion criteria was applied.

#### AX-CPT

Reactive and proactive control were measured using a child-friendly version of the AX-CPT paradigm (Chatham, Frank, & Munakata, 2009). The task was introduced as the Fruit Island game. An A or B cue (i.e. dog or cat) were presented in the middle of the screen for 500 ms followed by an inter-stimulus interval of 750 ms and then a probe X or Y (orange or apple) during which participants had to make their response. Participants had a maximum of 6000 ms to make a response. Participants were instructed to press the left key whenever an X followed an A (i.e. AX trials) and to press the down arrow key for the presentation of all other cue-probe combinations. Importantly, they were instructed to only respond once the probe had been presented and were alerted of this if they made a response before the probe was presented. Responses were followed by an inter-trial interval of 1,500 ms. The proportions of the trial types were based on Richmond et al. (2015) where 40% of trials were AX trials. All other trials (i.e. AY, BX, BY trials) were presented 20% each. Trials were presented randomly. 10 practice trials were administered where feedback was provided followed by 60 main trials. No exclusion criteria was applied.

### Statistical Analysis

#### Training Data

For the response inhibition, measures of mean SSRT, SSD and reaction times were calculated using the integration method (Verbruggen, Chambers, & Logan, 2013; Verbruggen et al., 2019). According to previous recommendations, rules were implemented in the calculation of the indices (Verbruggen et al., 2019; *Supplementary Materials*). For the context monitoring group, reaction times on correct context monitoring trials (corrRTCM) and Context Monitoring Signal delay (CMSD) were calculated. Training success was measured based on the slope of mean SSRT and corrRTCM for response inhibition and context monitoring groups respectively. For the response speed group main outcome measures were Correct Go RT (CorrGoRT) and the duration of stimulus presentation (StimDur) as a measure of adaptive difficulty in the task similar to the signal delay. Reaction times were included that were within 2 standard deviations of the mean reaction time per participant. Stimulus presentation durations of more than 10 seconds in length were excluded as these indicate performance on the games was not normal. Full information on sessions and data cleaning can be found in the *Supplementary Materials*.

We used multilevel modelling with sessions at the first level and participants at the second level, and our outcome measures (i.e. mean SSRT, SSD, reaction time) as the dependent measure using the lme4 package in R (Berkman et al., 2014). We investigated whether changes in the dependent measures over sessions for participant were better explained by a null model (model0 = Dependent Measure (DM) ~ 1 + (1|Participant)), a model with random intercept and fixed slope (model1 = DM ~ Session + (1|Participant)), or a model with a random intercept and slope per participant (model2 = DM ~ Session + (1 + Session|Participant)). Model fits were compared with a chi square test, and results from the best fitting model are reported in the results (for further information on the multilevel modelling see *Supplementary Materials*). Package lmerTest in R was used to acquire p-values (Kuznetsova, Brockhoff & Christensen, 2017) and confidence intervals were computed using bootstrapping, via the package boot in R (Ripley, 2020; Davison & Hinkley, 1997).

#### Pre-Post Tasks

To examine any changes in our measures pre- and post-training, repeated measures analyses were performed using mixed model ANOVAs with time point as a within-subject-factor and training group as a between-subject factor. Any significant interactions were further explored using paired sample t-tests.

#### Stop-Signal Response Task

Based on previous guidelines, reaction times below 100 ms and above 5000 ms were excluded (Luce, 1986). To analyse response inhibition derived from the SSRT, we calculated a *mean SSRT* estimate using the integration method (Verbruggen et al., 2013; Verbruggen et al., 2019). Along with this, we used measures of mean SSD and correct inhibition (%) to measure response inhibition.

#### AX-CPT

To obtain a measure of proactive control, we examined the difference between AY and BX trials for both reaction times and error rates. Using this method, a larger value indicates tendency to employ proactive rather than reactive control while a smaller value indicates tendency to employ reactive rather than proactive control.

## Results

### Training motivation and adherence

There was no significant difference in the total number of sessions attempted between the groups, (response inhibition group: *M* = 7.84, *SD* = 3.45; context monitoring group: *M* = 9.81, *SD* = 5.92; response speed group: *M* = 9.50, *SD* = 5.31, *F* (2,57) = .86, *p* = .428, *95% CI* [–1.58, 4.89]). After applying the exclusion criteria for sessions (see *Supplementary Materials*), there was still no significant difference between the groups for sessions included for analysis, (response inhibition group: *M* = 5.74, *SD* = 2.18; context monitoring group: *M* = 7.00, *SD* = 3.55; response speed group: *M* = 7.74, *SD* = 4.82, *F* (2,55) = 1.44, *p* = .246, *95% CI* [–.39, 4.39]).

There was no significant difference between the groups in the percentage of bonus games completed for the total number of sessions, (response inhibition group: *M* = 33.03, *SD* = 19.81; context monitoring group: *M* = 42.45, *SD* = 23.37; response speed group: *M* = 44.39, *SD* = 23.79; *F* (2,57) = 1.42, *p* = .250, *95% CI* [–3.05, 25.76]). There was also no difference in motivation scores over time between the groups (response inhibition: *M* = 62.98, *SD* = 30.90; context monitoring: *M* = 72.19, *SD* = 32.68; response speed: *M* = 71.09, *SD* = 36.44; *F* (2,212) = 1.63, *p* = .199, *95% CI* [–2.94, 19.28]).

### Response inhibition group

**Figure 1:**
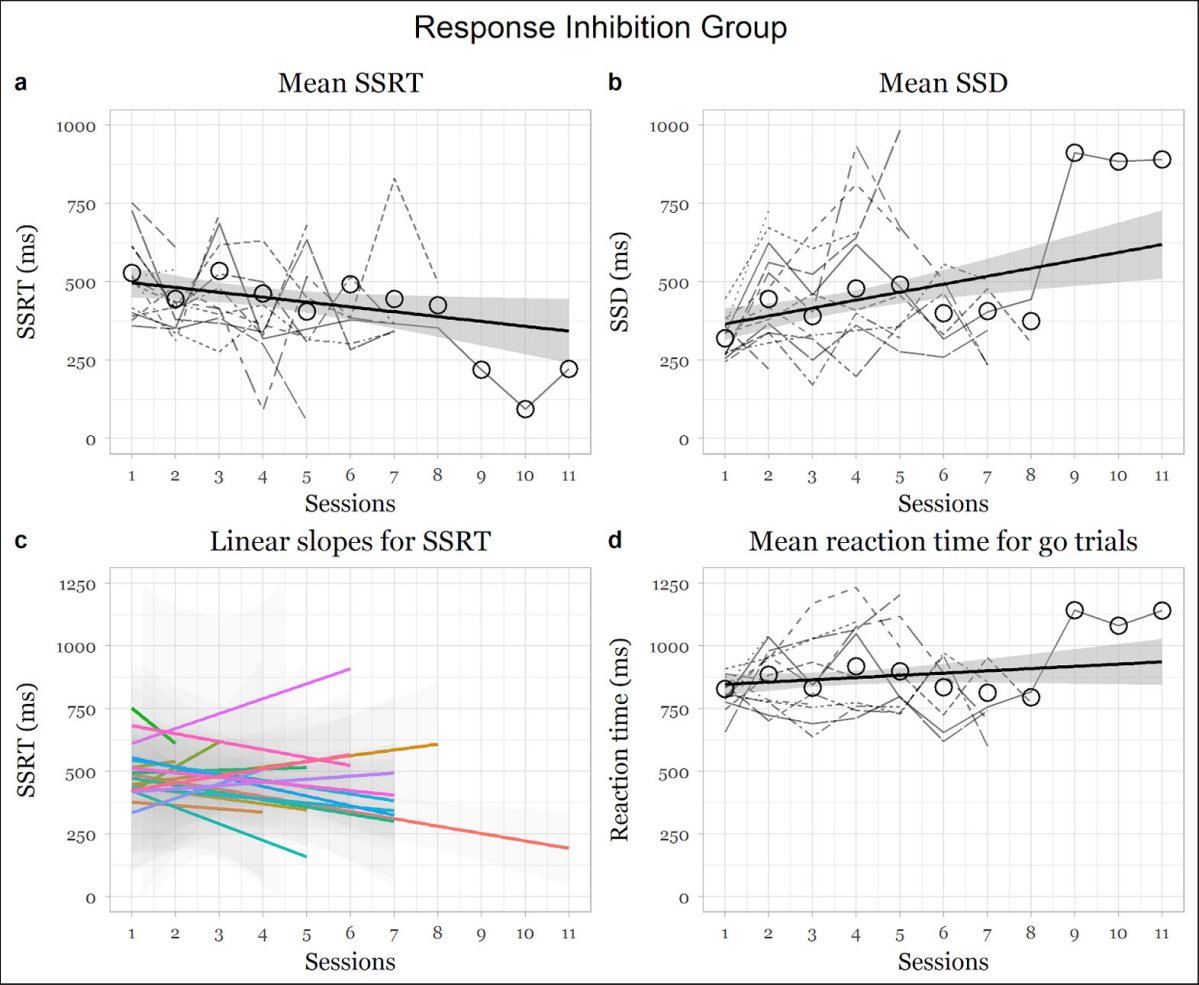
Changes in measures as a function of training in the response inhibition group. *Note*: Dotted and coloured lines indicate individual participant changes in the respective indices. Thick black line indicates average change over the weeks over all participants.

There was a significant negative main effect of session on SSRT, showing that the response inhibition group became significantly better at inhibiting over sessions, (*F* (1,254.4) = 5.97, *p* = .015, *95% CI* [–32.43, –3.16]; slope for session: *beta* = –18.30, *t* = –2.44, *se* = 7.49; Figure 1). There was a significant increase in the mean SSD values over sessions, (*F* (1,257) = 16.45, *p* < .001, *95% CI* [14.20, 40.18]; individual slope for session: *beta* = 27.68, *t* = 4.01, *se* = 6.83). There was also a significant increase in reaction time over sessions (*F* (1,256.41) = 4.88, *p* = .028, *95% CI* [2.00, 20.60]; slope for session: *beta* = 11.21, *t* = 2.21, *se* = 5.07).

### Context monitoring group

**Figure 2:**
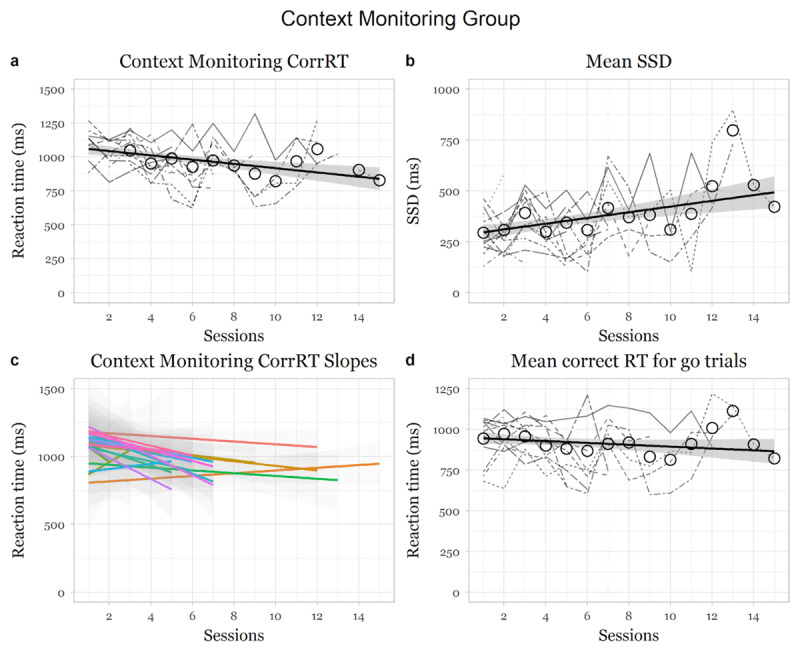
Changes in measures as a function of training in the context monitoring group. *Note*: Dotted and coloured lines indicate individual participant changes in the respective indices. Thick black line indicates average change over the weeks over all participants.

There was a significant main effect of session on corrRTCM, showing that the context monitoring group became significantly faster at correctly answering context monitoring trials over sessions, (*F* (1,220.71) = 8.46, *p* = .004, *95% CI* [–21.32, –4.06]; slope for session: *beta* = –12.44, *t* = –2.91, *se* = 4.28; Figure 2). There was a significant increase in the mean CMSD values over sessions, (*F* (1,250.98) = 15.24, *p* < .001, *95% CI* [7.29, 22.71]; individual slope for session: *beta* = 14.76, *t* = 3.90, *se* = 3.78; Figure 2), showing that the participants became better at the task. There was no significant change in CorrGoRT over sessions (*F* (1,234.24) = 0.69, *p* = .408, *95% CI* [–10.72, 5.12]; slope for session: *beta* = –3.31, *t* = –0.83, *se* = 4.00; Figure 2).

### Response speed group

**Figure 3:**
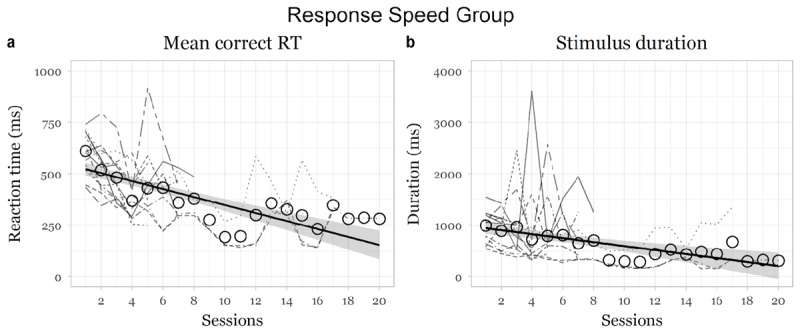
Changes in measures as a function of training in the context monitoring group. *Note*: Dotted lines indicate individual participant changes in the respective indices Thick black line indicates average change over the weeks over all participants.

There was a significant main negative effect of session on CorrGoRT, showing that the response speed group became significantly faster over sessions, (*F* (1,306.33) = 49.76, *p* < .001, *95% CI* [–21.19, –12.23]; slope for session: *beta* = –16.61, *t* = –7.05, *se* = 2.35; Figure 3). StimDur significantly decreased over sessions, (*F* (1,303.1) = 4.62, *p* = .032, *95% CI* [–37.92, –1.48]; slope for session: *beta* = 19.62, *t* = –2.15, *se* = 9.13; Figure 3), showing that the participants improved at the tasks over sessions.

#### Baseline indices

**Figure 4:**
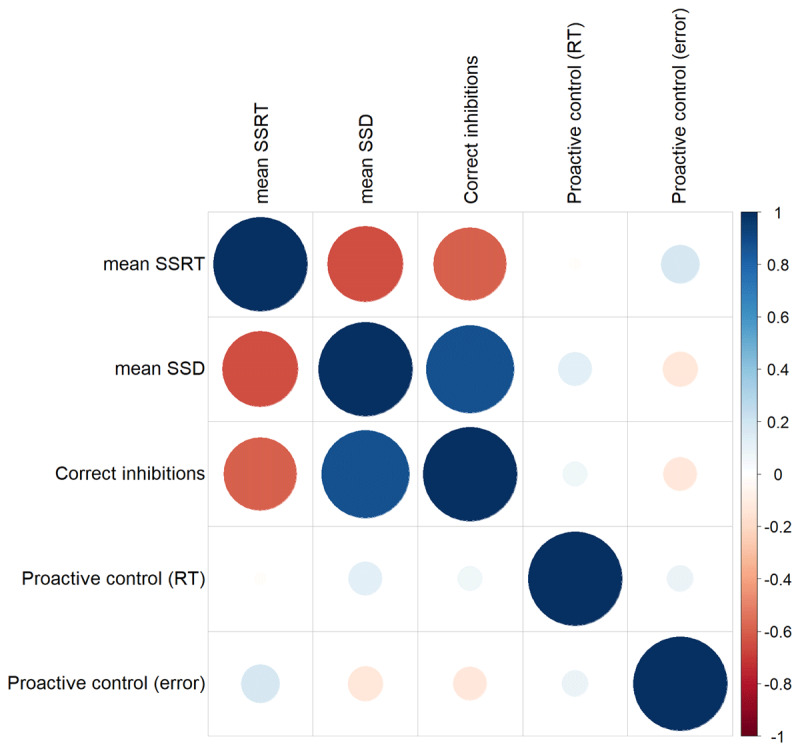
Correlations between measures of interest have been shown.

No differences in baseline scores in mean SSRT, mean SSD, correct inhibition and proactive control scores were observed (*t* < 2.05, *p* > .05). Correlations between these indices have been shown in Figure 4.

#### Pre- and Post-Training Response inhibition

**Figure 5:**
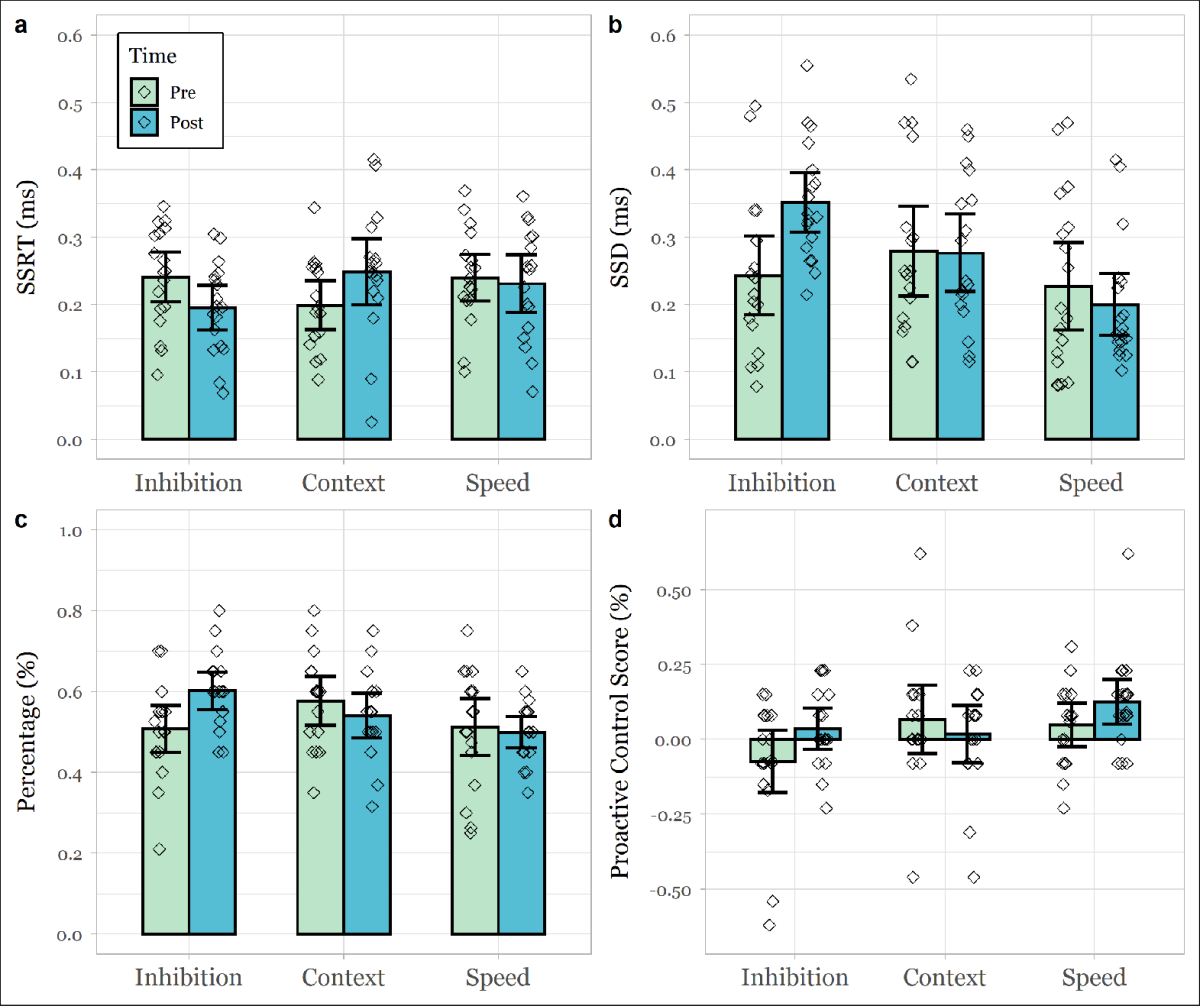
Pre-post test changes of mean SSRT, SSD, correct percentage inhibitions and proactive control (%) in all three groups.

Using all three measures of response inhibition (i.e. mean SSRT, mean SSD, correct inhibitions) as a criterion showed significant interactions between group and timepoint (Tables 2, 3, 4, 7). To further examine these interactions, paired t-tests were used to investigate the effect of training in the different groups. For the response inhibition group there was a significant reduction in mean SSRT (*t* (17) = 2.10, *p* = .05), a significant increase in mean SSD (*t* (17) = –3.62, *p* = .02) and a significant increase in correct inhibitions (%) (*t* (17) = –3.01, *p* = .01) between pre- and post-training (Figure 5A, 5B, 5C). For the context monitoring group, there was a significant increase in mean SSRT (*t* (19) = 2.24, *p* = .04), but no significant change for mean SSD (*t* (19) = –0.47, *p* = .65) and correct inhibitions (%) (*t* (19) = 0.17, *p* = .87) between pre- and post-training (Figure 5A, 5B, 5C). For the response speed group, there were no significant differences in mean SSRT (*t* (17) = 1.06, *p* = .30), mean SSD (*t* (17) = 0.79, *p* = .44) or correct inhibitions (%) (*t* (17) = 0.30, *p* = .77) between pre- and post-training (Figure 5A, 5B, 5C). Because we were specifically interested in the effects of the different types of cognitive control training on outcome measures, we also compared these two groups directly. There were significant pre-post differences in all three measures between response inhibition and context monitoring groups (Table 8), where the response inhibition group benefited significantly more from the training than the context monitoring group.

**Table 2:** Results from Mixed ANOVA examining mean SSRT.


PREDICTOR	*DF_NUM_*	*DF_DEN_*	*F*	*P*	η^2^

(Intercept)	1	54	609.34	.000	0.92

Group	2	54	0.34	.713	0.011

Timepoint	1	54	0.11	.742	0.00

Group × Timepoint	2	54	5.18	.009	0.16


**Table 3:** Results from Mixed ANOVA examining mean SSD.


PREDICTOR	*DF_NUM_*	*DF_DEN_*	*F*	*P*	η^2^

(Intercept)	1	54	399.31	.000	0.88

Group	2	54	3.52	.037	0.12

Timepoint	1	54	3.97	.051	0.07

Group × Timepoint	2	54	5.68	.006	0.17


**Table 4:** Results from Mixed ANOVA examining correct inhibitions (%).


PREDICTOR	*DF_NUM_*	*DF_DEN_*	*F*	*P*	η^2^

(Intercept)	1	54	1643.12	.000	0.97

Group	2	54	1.11	.336	0.04

Timepoint	1	54	2.49	.121	0.04

Group × Timepoint	2	54	3.83	.028	0.12


None of our cognitive control measures at pre-test predicted training success in either response inhibition or context monitoring groups (p > .114). Training success did not predict changes in mean SSRT in either group (*p* > .153).

#### Proactive Control

We examined how proactive control changes with training. Analysis revealed a non-significant interaction between group and timepoint for proactive control based on reaction time (Table 5). There was a marginally significant interaction between group and timepoint for proactive control based on error rates (Table 6). To further examine this trend, paired t-tests were used to investigate the effect of training in the different groups. As predicted, in the response inhibition group, proactive score (%) significantly increased between pre- and post-training (*t* (16) = –2.60, *p* = .019). There were no significant differences in proactive score (%) in either the context monitoring (*t* (15) = 0.96, *p* = .35) or response speed groups (*t* (16) = -1.29, *p* = .21) between pre- and post-training (Figure 5D). We also compared the two cognitive control training groups on this outcome measure and found a significant difference between pre-post changes in proactive score (%) between the response inhibition and context monitoring group (Table 7). Proactive control measures at pre-test were not associated with training success (*p* > .587) in either response inhibition or context monitoring group. In neither training group, did training success predict changes in proactive control score (*p* > .332).

**Table 5:** Results from Mixed ANOVA examining proactive control score (s) based on reaction times.


PREDICTOR	*DF_NUM_*	*DF_DEN_*	*F*	*P*	η^2^

(Intercept)	1	53	17.37	.586	0.25

Group	2	53	1.75	.407	0.06

Timepoint	1	53	0.58	.647	0.01

Group × Timepoint	2	53	0.75	.241	0.03


**Table 6:** Results from Mixed ANOVA examining proactive control score (%) based on error rates.


PREDICTOR	*DF_NUM_*	*DF_DEN_*	*F*	*P*	η^2^

(Intercept)	1	53	4.37	.887	0.08

Group	2	53	2.31	.188	0.08

Timepoint	1	53	2.49	.211	0.04

Group × Timepoint	2	53	2.93	.064	0.10


**Table 7:** Comparisons from independent t-tests of response inhibition and context monitoring groups based on pre-post changes in cognitive control indices.


	*RESPONSE INHIBITION GROUP*		*CONTEXT MONITORING GROUP*		*RESPONSE SPEED GROUP*	
		
	M (SD)		M (SD)		M (SD)	
		
	PRE-TEST	POST-TEST	PRE-TEST	POST-TEST	PRE-TEST	POST-TEST

mean SSRT (s)	0.24 (0.07)	0.20 (0.07)	0.22 (0.08)	0.27 (0.10)	0.24 (0.07)	0.23 (0.08)

mean SSD (s)	0.24 (0.12)	0.35 (0.09)	0.25 (0.14)	0.26 (0.11)	0.22 (0.13)	0.20 (0.09)

Correct inhibitions (%)	0.51 (0.12)	0.60 (0.09)	0.53 (0.16)	0.53 (0.10)	0.51 (0.14)	0.50 (0.08)

Proactive control (%)	–0.07 (0.21)	0.04 (0.13)	0.07 (0.22)	0.02 (0.19)	0.05 (0.14)	0.13 (0.16)


**Table 8:** Comparisons from independent t-tests of response inhibition and context monitoring groups based on pre-post changes in cognitive control indices.


	*RESPONSE INHIBITION GROUP*	*CONTEXT MONITORING GROUP*		
	
	M (SD)	M (SD)	t-STATISTIC	p-VALUE

mean SSRT (s)	–0.05 (0.09)	0.06 (0.12)	–3.03	.004

mean SSD (s)	0.11 (0.03)	0.01 (0.11)	2.48	.018

Correct inhibitions (%)	0.09 (0.13)	–.00 (0.12)	2.43	.020

Proactive control (%)	0.12 (0.19)	–0.06 (0.26)	2.31	.027


*Note*: Decrease in mean SSRT indicated increase in cognitive control. Increases in mean SSD, correct inhibitions and proactive control scores indicated increase in cognitive control.

#### Effect of age

Training changes were not associated with age for any of the measures (*r* < .14, *p* > .306).

## Discussion

This study addressed the nature of processes underlying cognitive control during childhood, namely response inhibition and context monitoring. To provide causal evidence we leveraged a randomized controlled trial design and compared how training different cognitive processes impact cognitive control. Training involved 6 weeks of practicing either response inhibition, context monitoring or response speed. All groups improved on the cognitive domain that was trained, demonstrating that the adaptive training was successful. Crucially, pre-post-test comparisons on several measures of cognitive control revealed that only inhibition training successfully improved cognitive control. These findings demonstrate that response inhibition (and not context monitoring) plays a privileged role in cognitive control during childhood. This helps to resolve a long-standing debate in the field and points towards fruitful directions in terms of interventions aiming to improve this crucial skill.

We employed an adaptive intervention to improve effectiveness of training (Cuenen et al., 2016), in a gamified format suitable for children. This ensured that training was adjusted to each individuals’ ability and that training success was therefore maximised in all training groups. This also suggests that all training groups are likely to equally benefit from training and any differences in training effects are unlikely to be pre-existing explained by individual differences. Finally, training was equally engaging for all groups with no reported differences in motivation. This helps to rule out any potential confounders of group differences (in terms of either training effectiveness or motivation) accounting for our observations.

We used this gold standard training design to investigate how specific training regimes lead to improvements in cognitive control, by measuring transfer to novel tasks. We showed that post-training improvements in cognitive control are only observed in the group training response inhibition. Our findings are buttressed by evidence of a shift from reactive to proactive control observed in the response inhibition group only. This finding is in line with a previous study in adults, which found that response inhibition training led to changes in brain activity indicative of greater proactive control following training (Berkman et al., 2014). Training response inhibition thus not only induces improvements during training but also transfers to other contexts. These findings taken together, suggest that response inhibition may have important role in the plasticity of cognitive control. By that we mean that response inhibition rather than context monitoring may be the more malleable process of cognitive control, in turn likely to lead to greater changes in transfer functions following periods of extended training. This reinforces the privileged role response inhibition may hold as compared to context monitoring.

Despite improvements of context monitoring abilities during training, this did not transfer to pre-post measures of cognitive control. In fact, we report a surprising decline on a pre-post measure of cognitive control after training in the context monitoring group. This contrasts with previous findings showing positive effects of context monitoring practice on cognitive control (Chevalier et al., 2014). Several reasons might account for these discrepant results. First, previously used pre-post test stimuli were similar to those used for practice (Chevalier et al., 2014). Thus, context monitoring may only improve cognitive control measures when both practice and outcome measures are based on similar stimuli, suggesting that practice of context monitoring may improve processing of cues specific to monitoring but not an underlying cognitive skill. Second, there might be a critical difference in the extent of time dedicated to improving the skill in question. While practicing both inhibition and context monitoring in the short term can enhance cognitive control (Chevalier, 2014), our study suggests that, training more extensively and over longer periods the effects of these training paradigms differentiate. Thus, over short periods of time, both response inhibition and context monitoring practise improve monitoring capacities, but over longer periods, the action (going vs stopping) becomes more impactful. This may have led to more inconsistent inhibitions when the stop signal is presented in the context monitoring group, leading to a decline in cognitive control post-test. This points towards key differences in the underlying mechanisms involved – where for example, in the short term, heightened responsiveness to cues may lead to an overall increased ability to respond to stimuli (leading to generally improved performance, including in a response inhibition task), which over the longer term, if this is not paired with motoric response inhibition, is not sufficient.

One potential issue that may warrant consideration is how generalisable our findings may be. We recruited and tested participants from three schools that were willing to be part of our study which may have potentially biased our results. Future findings should recruit from a range of different schools with more diverse demographics. Further, we note that this is a pilot study, a first step in understanding cognitive control training. Therefore, future studies should further investigate the plasticity of cognitive control training through inhibition in bigger samples. Another limitation our study is that cognitive control (i.e. inhibition) was solely measured using the SSRT task. This may mean that any transfer observed may be task specific. Ideally, future research should examine transfer into cognitive control through a range of inhibition tasks (e.g. Stroop, flanker inhibition tasks).

Despite this, our study contributes to the cognitive control field significantly. Training can be costly and therefore, it is important to establish the mechanism underlying cognitive control. Our findings suggest that the type of mechanism targeted by interventions is not a trivial matter and may produce different changes in cognitive control. This is important as recommendations for training based on mechanism will differ as well – where inhibition may target improving ability to stop actions, context monitoring focuses on broadening attentional focus (Messer et al., 2018; Chatham et al., 2012). Importantly, it is crucial for researchers to adopt a clear framework when considering training cognitive control as this may help boost the effectiveness of training (Smid, Karbach, & Steinbeis, 2020). In particular, after establishing the mechanism underlying training, it may be important to examine individual differences that may predict training and timepoints at which training may be most effective. Perhaps, some of these correlates as well as other pre-existing individual characteristics could even give us insight into variability observed in training success (Konen & Karbach, 2015).

The present study is the first to use a gamified, adaptive and variable training intervention in a randomized control trial to causally test the contributions of response inhibition and context monitoring to cognitive control in childhood. Only the inhibition group improved on post-training measures of cognitive control. These findings help to resolve the debate around the key mechanisms facilitating cognitive control, suggesting that inhibition may have a privileged role in cognitive control during childhood. Inhibition training interventions such as the one used in the current study hold promise for improving cognitive control at developmental periods of heightened plasticity. These findings are of note given that childhood cognitive control is predictive of later life success and well-being.

## Data Accessibility Statement

Neither of the experiments reported in this article was formally preregistered. Data will be made available at github.com/dcp-lab.

## Additional File

The additional file for this article can be found as follows:

10.5334/joc.314.s1 Supplementary Materials. Further information on training paradigm.
