# Supplementary material for: EXAMINING MECHANISMS OF CHILDHOOD COGNITIVE CONTROL
Source: J Cogn. 2023 Aug 25;6(1):50. doi: 10.5334/joc.314 (PMC10453963; doi:10.5334/joc.314)
Supplement: Supplementary Materials. — Further information on training paradigm. [file joc-6-1-314-s1.pdf]

Supplementary Materials

Information on training games.

Six training games were available for each group. These were Treasure collect, Mining, Chest picking, Conveyor belt, AB driving and Hold-and-Release (HR) driving (see Supplementary Figure 1). Each of these had 4 different settings (forest, desert, snow, mountain) which was randomly assigned based on an assignment table. The games playable varied per session. Participants were initially presented with a selection of caves that they could choose from before starting their game to encourage engagement (Johann & Karbach, 2020). For each game, the participant was presented with a narrative in which they were required to accrue points by collecting treasure, gems, or coins, whilst avoiding a perpetrator (dragon, monster, ghost). For all the games, participants were instructed to respond in a particular way to a certain stimulus, and respond in a different way to a different stimulus in order to collect points. These instructions varied in different ways across the three training groups, in order to train the targeted abilities (Table 1). For example, the three groups were given the following instructions for the Treasure Collector game:

- Response inhibition training group: “You will see a pile of treasure. Collect it by pressing ‘space’. A dragon is guarding the treasure. When you see it, do NOT press ‘space’. Just wait for the dragon to go away, and you will win a gem!”.
- Context monitoring training group: “You will see a pile of treasure. Collect it by pressing ‘space’. A dragon is guarding the treasure. When you see it press ‘space, space’ to make the dragon to go away and you will win a gem!”
- Response Speed training: “You will see a pile of treasure. Collect it by pressing ‘space’. A dragon is guarding the treasure. When you see it, that

means there is a precious gem in the treasure. Press ‘space’ as fast as you can to collect the gem!”

Training data cleaning process

As these games were being piloted and development of the games was still not entirely completed, there was a small percentage of errors in the raw data in the form of invalid key presses (<5%), incorrect coding of separate sessions by date, or session durations that went on too long (<5%). In order to accurately analyse the training data, invalid presses for the games were removed and sessions were trimmed if they continued on too long (15 minute duration per session). If more than 50% of trials were removed, the game was excluded from analysis. For SSRT calculation (where applicable), it was ensured that stop RT was larger than go RT and any negative SSRT values were removed (Verbruggen, 2019).

Below we will outline the game mechanisms per group:

Response inhibition group. For the Treasure collect, Mining, Chest picking and Conveyor belt game, valid responses were either spacebar presses or no responses. Any different key presses that were logged in the raw data (e.g. arrow key or enter presses) were excluded from the data (<5%). For the AB driving game, valid responses were the left and right arrow key, or no response. Any spacebar presses or other keys were excluded from the data (<5%). For the HR driving game, valid responses were spacebar release and no responses. Any other responses were excluded from the data.

Context monitoring group. For the treasure collect, mining, chest picking and conveyor belt game and HR driving, valid responses were a single spacebar press, spacebar release, a double spacebar press or a no response. All arrow key responses (<5%) were excluded from the data. For the AB driving game, left, right and up arrow key responses and a 'none' response were valid responses. All others were excluded from the data (<5%).

Response speed group. For the treasure collect, mining, chest picking and conveyor belt game and HR driving, valid responses were a spacebar press or a no response. All arrow key responses were excluded from the data (<5%). For the AB driving game, valid responses were left and right arrow keys or no responses. All other keys were excluded from the data (<5%).

Session recoding and inclusion

For all groups, sessions were recoded based on date, meaning any data logged on the same date would be grouped in the same session. Since the mechanisms of the games differed in terms of key presses and mechanisms tested (see training game tasks), we only included sessions for a participant that a) had a minimum of 2 games (e.g. if the session only consisted of one game it was not included in the mixed model analysis), and for the response inhibition group only, sessions that b) that had at least two games which both had valid (e.g. not negative or an SSRT value that could not be calculated) SSRT measures. For the response speed group, reaction times were included that were within 2 standard deviations of the mean reaction time per participant. Stimulus duration was adaptive on participant performance, and a stimulus duration of over 10 seconds meant that the participant

reacted extremely slow. All further analysis for the training data for all three groups only included sessions that were not excluded based on these terms (e.g. also for the behavioural data analysis regarding accuracy).

Motivation Questionnaire. The questionnaire consisted of 6 items (1*. I like the training*; 2*. I like to do the training*; 3*. I do not always feel like training*; 4*. I think I can become better through the training*; 5*. I find the training boring*; 6*. I am getting better at the training tasks)*. Items 3 and 5 were reverse coded.

Modelling for training measures

Response Inhibition group. First, we investigated whether change in SSRT over sessions for the participants was better explained by a null model (model0 = SSRT ~ 1 + (1 | Participant)), a linear model with random intercept and fixed slope (model1 = SSRT ~ Session + (1 | Participant)), or a linear model with a random intercept and slope per participant (model2 = SSRT ~ Session + (1 + Session | Participant)). A chi square test showed that model1, (-2LL = 3578.3, where −2LL is −2 * log-likelihood (or the deviance parameter reported in lme4, (Bates, 2010)), AIC = 3586.3, BIC = 3600.5) fit significantly better than model2, (2LL = 3576.9, AIC = 3588.9, BIC = 3610.2 ; X^2 = 5.873, p = .015) and the null model. We therefore used model1 for SSRT and sessions to report the following results for the response inhibition group. A chi square test showed that model1, (-2LL = 3529.5, AIC = 3537.5, BIC = 3551.7), also fit significantly better for SSD than model2, (-2LL = 3556.4, AIC = 3535.1, BIC = 3556.4; X^2 = 15.93, p = < .001) and the null model, so we used model1 to investigate changes in SSD over sessions. For reaction time on correct go trials (CorrGoRT), a chi-square test showed that model2, (-2LL = 3368.0,

AIC = 3380.0, BIC = 3401.3), fit significantly better than model1 (-2LL = 3377.5, AIC = 3385.5, BIC = 3399.6; X^2 = 9.41, p = .009). However, fitting model2 led to a singular fit, probably due to the random effects structure leading to overfitting of the data, so we used model1 instead to report the following results.

Context monitoring group. First, we investigated whether change in correct RT for context monitoring trials (corrRTCM) over sessions for the participants was better explained by a null model (model0 = corrRTCM~ 1 + (1 | Participant)), a linear model with random intercept and fixed slope (model1 = corrRTCM~ Session + (1 | Participant)), or a linear model with a random intercept and slope per participant (model2 = corrRTCM~ Session + (1 + Session | Participant)). A chi square test showed that model1, (-2LL = 4434.2, AIC = 4442.2, BIC = 4457.3) fit significantly better than model2, (2LL = 4433.6, AIC = 4445.6, BIC = 4468.2 ; X^2 = 8.21, p = .004) and the null model. We therefore used model1 for corrRTCM and sessions to report the following results for the context monitoring group.For SSD, a chi square test showed that model1, (-2LL = 4450.2, AIC = 4458.2, BIC = 4473.4), also fit significantly better for SSD than model2, (-2LL = 4449.9, AIC = 4461.9, BIC = 4484.7; X^2 = 14.53, p < .001) and the null model, so we used model1 to investigate changes in SSD over sessions.For CorrGoRT, none of the models fit the data significantly better than the null model, and we therefore assume there was no change in CorrGoRT over sessions. We still reported these non-significant effects with the next best fitting model. The chi-square test showed that model2 was the better fit, (-2LL = 4492.4, AIC = 4504.4, BIC = 4527.2), but not significantly more than model1 (-2LL = 4496.5, AIC = 4504.5, BIC = 4519.6; X^2 = 4.03, p = .134). We used model2 to report the results.

Response speed group. We tested whether CorrGoRT over sessions for the participants was better explained by a null model (model0 = CorrGoRT ~ 1 + (1 | Participant)), a linear model with random intercept and fixed slope (model1 = CorrGoRT ~ Session + (1 | Participant)), or a linear model with a random intercept and slope per participant (model2 = CorrGoRT ~ Session + (1 + Session | Participant)). A chi square test showed that model1, (-2LL = 3983.9, AIC = 3991.9, BIC = 4006.8) fit significantly better than model2, (2LL = 3982.3, AIC = 3994.3, BIC = 4016.6 ; $X^2 = 45.76$, $p < .001$) and the null model. For StimDur, model1 was the best fit for the data, (-2LL = 4819.9, AIC = 4827.9, BIC = 4842.8) when compared to model2, (-2LL = 4819.7, AIC = 4831.7, BIC = 4854.1; $X^2 = 4.46$, $p = .035$) and the null model.

Table 1. Table of stimulus-response instructions for each game, across the different training groups.

Game	Stimulus	Response inhibition	Context Monitoring	Response Speed
Treasure Collector	1) Treasure	Press space (go)	Press space	Press space
	2) Dragon	Do not press space (stop)	Double press space	Press space
Mining	1) Rock	Press space (go)	Press space	Press space
	2) Gem	Do not press space (stop)	Double press space	Press space
Chest picking	1) Wobbling treasure chest on the other side to your bag	Press space to move to other side (go)	Press space to move to other side	Press space to move to other side
	2) Wobbling treasure chest on the same side as	Do not press space (stop)	Double press space	Do not press space

	your bag			
	3) Dragon	Press space to move away from the dragon (go). Do not press space if the dragon is on the other side (stop).	Double press space to avoid moving towards the dragon	Press space to move underneath the dragon
Conveyor belt	1) Wobbling treasure chest	Press space to change direction of the belt so that the treasure chest moves towards the bag (go).	Press space to change direction of the belt. Double press space to keep the same direction.	Press space to change direction of the belt so that the treasure chest moves towards the bag.
	2) Dragon	Avoid the chest with the dragon behind it by pressing space to change the direction (stop).	Avoid the chest with the dragon behind it by pressing space to change the direction. Double press space to keep same direction.	Move the chest with dragon behind it by pressing space to change the direction.
AB Driving	1) Sign pointing left or right	‘Left’ or ‘Right’ arrow key (go)	‘Left’ or ‘Right’ arrow key	‘Left’ or ‘Right’ arrow key
	2) Stop sign	Do not press ‘left’ or ‘right’ arrow key (stop)	Press ‘upwards’ arrow key	Press ‘left’ or ‘right’ arrow key
HR Driving	*	Continuously hold space (go)	Continuously hold space	Continuously hold space
	1) Ghost looking at the front of your car	Stop holding space (stop)	Stop holding space	Stop holding space
	2) Ghost looking at back of your car	Keep holding space (go)	Keep holding space and press enter	Stop holding space

*In this game, the instructions are the opposite in that the participants are required to continuously hold space and *stop* holding space in response to certain stimuli.

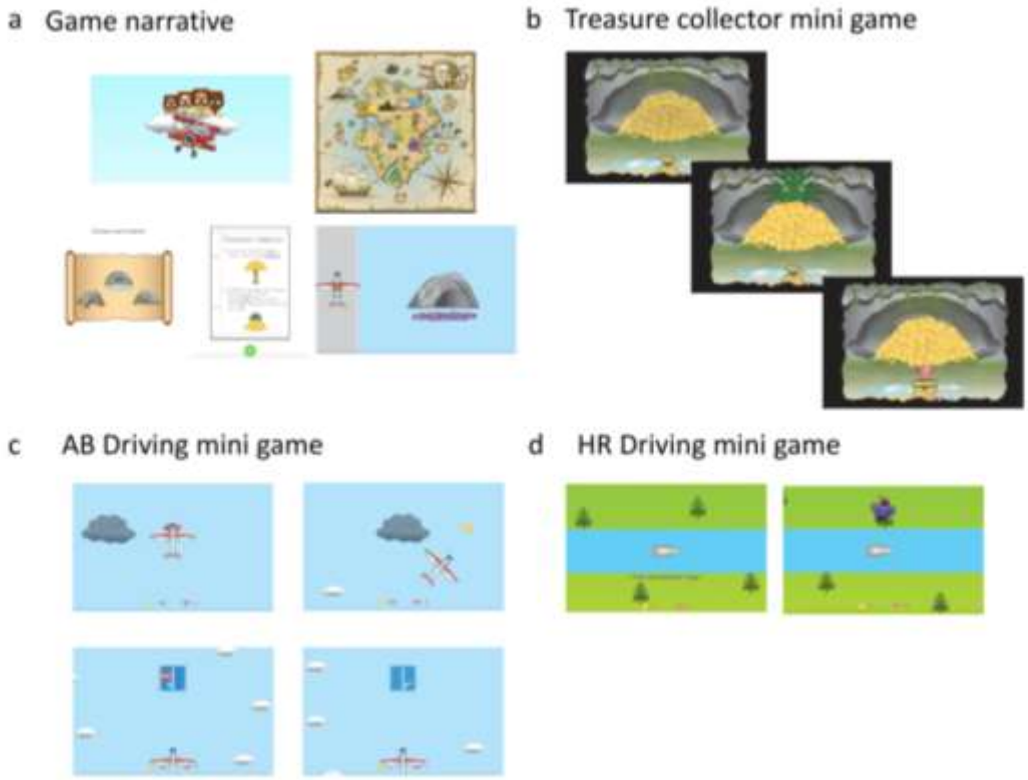

Supplementary Figure 1A. An illustration of the overall narrative of the games, B. Example images of the Treasure Collector game, C. Example images of the AB Driving game, D. Example images of the HR Driving game.
